# Antibiotic Susceptibility of Potentially Probiotic Vaginal Lactobacilli

**DOI:** 10.1155/IDOG/2006/18182

**Published:** 2006-10-01

**Authors:** Virginia Ocaña, Clara Silva, María Elena Nader-Macías

**Affiliations:** ^1^Nuevo Hospital El Milagro, Salta 4400, Argentina; ^2^Facultad de Bioquímica, Química y Farmacia, Universidad Nacional de Tucumán, Tucumán 4000, Argentina; ^3^Centro de Referencia para Lactobacilos (CERELA-CONICET), Chacabuco 145, Tucumán 4000, Argentina

## Abstract

*Objective*. To study the antimicrobial susceptibility of six vaginal probiotic lactobacilli. *Methods*. The disc diffusion method in Müeller Hinton, LAPTg and MRS agars by the NCCLS (National Committee for Clinical Laboratory Standards) procedure was performed. Due to the absence of a *Lactobacillus* reference strains, the results were compared to those of *Staphylococcus aureus* ATCC29213. Minimal Inhibitory Concentration (MIC) with 21 different antibiotics in LAPTg agar and broth was also determined. *Results*. LAPTg and MRS agars are suitable media to study antimicrobial susceptibility of lactobacilli. However, the NCCLS procedure needs to be standardized for this genus. The MICs have shown that all *Lactobacillus* strains grew at concentrations above 10 *μ*g/mL of chloramphenicol, aztreonam, norfloxacin, ciprofloxacin, ceftazidime, ceftriaxone, streptomycin and kanamycin. Four lactobacilli were sensitive to 1 *μ*g/mL vancomycin and all of them were resistant to 1000 *μ*g/mL of metronidazole. Sensitivity to other antibiotics depended on each particular strain. *Conclusions*. The NCCLS method needs to be standardized in an appropriate medium to determine the antimicrobial susceptibility of *Lactobacillus*. Vaginal probiotic lactobacilli do not display uniform susceptibility to antibiotics. Resistance to high concentrations of metronidazole suggests that lactobacilli could be simultaneously used with a bacterial vaginosis treatment to restore the vaginal normal flora.

## INTRODUCTION

Bacteria of the genus *Lactobacillus* have been proposed as probiotic microorganisms to restore the ecological equilibrium of the intestinal, respiratory, and urogenital tracts [1]. This type of bacterial replacement therapy has been widely used as fermented milks to prevent diarrhea in humans and animals [2, 3]. They have also been increasingly considered for their use in women to prevent genital and urinary tract infections [4–8].

It has been found that administration of antimicrobial substances alters the microbial balance of the vagina and suppresses certain bacterial groups [4]. The effect of these substances on autochthonous *Lactobacillus* is of interest in understanding the development of genital and urinary tract infections related with the lack of these bacteria [9].

The present study was conducted to determine the antimicrobial susceptibility of six candidate probiotic *Lactobacillus* strains. These lactobacilli have been previously selected for probiotic properties as surface hydrophobicity [10], self-and coaggregation [11], adhesion to vaginal epithelial cells [12], and production of antimicrobial substances [13–15]. The main aims of knowing the behavior of exogenously applied *Lactobacillus* under the effect of antimicrobial substances are to have an approach of the response of lactobacilli administered to patients subjected to some kind of antibiotic therapy and to consider the concomitant use of lactobacilli and an antibiotic to restore the disrupted ecological environment.

Having in mind that a method to study antimicrobial susceptibility of genus *Lactobacillus* has not been standardized yet, different techniques were assayed. The results obtained by using the disc diffusion method with culture media different from Müller Hinton agar proposed by the NCCLS (National Committee for Clinical Laboratory Standards) and the determination of the minimal inhibitory concentrations in an enriched medium are described in this paper.

## MATERIALS AND METHODS

### Microorganisms and growth conditions

The microorganisms used in this study were *Lactobacillus acidophilus* CRL1251 (Centro de Referencia para Lactobacilos Culture Collection), *Lactobacillus paracasei ssp paracasei* CRL1289, *Lacidophilus* CRL1266 (H_2_0_2_-generating strains), *L gasseri* CRL1259 (organic acid producer), *L johnsonii* CRL1294 (aggregating), and *L salivarius* CRL1328 (bacteriocin producer). They have been isolated from the human vagina of women from Tucumán, Argentina, and identified by biochemical profiles, sugar fermentation patterns, and API 50 system (BioMérieux Vitec, Inc, France) [10]. NCCLS type strain, *Staphylococcus aureus* ATCC29213 from the American Type Culture Collection, was employed as reference strain.

All the microorganisms were stored in milk-yeast extract at −70°C. Prior to the assays, they were subcultured twice in LAPTg broth [16], and a third time in the media where the susceptibility to antibiotics assay was going to be performed: MRS [17], LAPTg, or Müller Hinton broth.

### Antimicrobial agents

Inhibitors of the cell wall synthesis (oxacillin, aminopenicillins, ceftazidime, ceftriaxone, cefotaxime, imipenem, aztreonam, and vancomycin), protein synthesis (kanamycin, gentamicin, streptomycin, tetracyclines, chloramphenicol, clarithromycin, erythromycin, and nitrofurantoin), and nucleic acid synthesis (trimethoprim-sulfamethoxazole, rifampin; norfloxacin, ciprofloxacin, nalidixic acid, pipemidic acid, and metronidazole) were employed for inhibition tests. They were used as commercial discs (Britania, Argentina) or prepared from drugs provided by different companies (Sigma, USA; Merck, Germany; Britania, Argentina; ICN, Argentina).

### Disc diffusion method

Antimicrobial susceptibility was studied by employing the method described by Bauer et al [18] for clinical isolates, modified by using three different base agar media: Müller Hinton, LAPTg, and MRS agars. Frozen microorganisms were subcultured twice in LAPTg broth and a third time in MRS, LAPTg, or Müller Hinton broth for 14 hours at 37°C. Suspensions were adjusted to tube 5 in McFarland scale (10^8^ CFU/mL) and the microorganisms were (a) disseminated on the surface of MRS, LAPTg, or Müller Hinton agar plates with embedded swabs and (b) included into the agar. To include the lactobacilli into the agar, 100 *μ*L of the microbial suspension were mixed with 12 mL of melted agar (melted and cooled down to 45°C) and then poured on plates. Antibiotic discs were placed on the surface of the agar (six discs in each plate) and the plates were incubated for 24 to 48 hours at 37°C under microaerophilic conditions. After the incubation, the diameter of the halos was measured.

### Minimal inhibitory concentrations

The MICs were determined in LAPTg broth and agar. Solutions of each antibiotic at concentrations of 10 to 50 mg/mL were prepared. They were serially diluted in LAPTg broth and added to LAPTg broth or 45°C melted agar to obtain final concentrations of 1 to 1000 *μ*g/mL. Fifty *μ*L of exponential growth phase microorganisms at concentration of 10^7^ to 10^8^ CFU/mL were inoculated in LAPTg with antibiotics. Cultures were incubated up to 48 hours at 37°C and the inhibition of growth was spectrophotometrically determined at 540 mn (Gilford Spectrophotometer, USA) for assays performed in LAPTg broth and by macroscopic observation for agar tests.

### Statistical evaluation

The disc diffusion method was performed by duplicate and the diameters obtained for each strain are represented in the tables. MIC test was performed by triplicate. Complete inhibition of growth in all three tubes or plaques with the same antibiotic concentration was considered as the MIC.

## RESULTS

### Disc diffusion method

**Figure 1:**
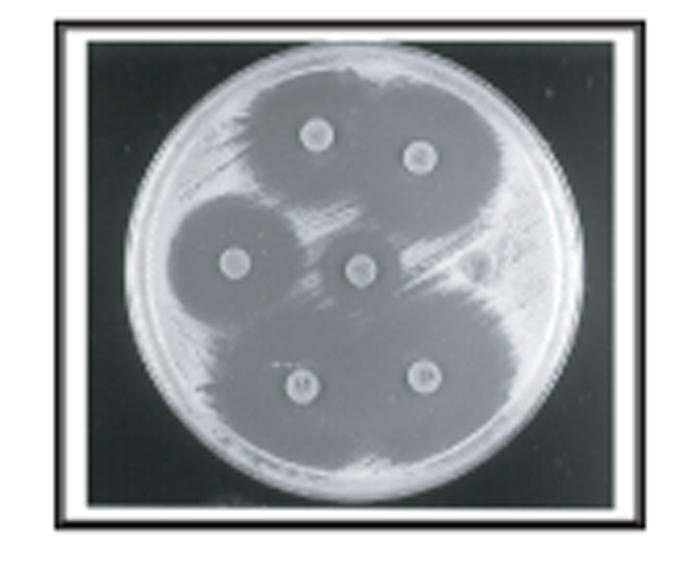
Semiquantitative disc assay developed in LAPTg agar for *Lactobacillus acidophilus* CRL1251 inoculated on the surface.

Growth of lactobacilli in Müller Hinton broth was poor and when any type of growth was detected on the agar, it was irregular and the halos were undefined. In LAPTg agar the inhibition halos were sharply defined (Figure 1) and the diameters could be easily measured when the microorganisms were inoculated either on the surface or into the agar. On the other side, the diameters of the halos for lactobacilli inoculated on the surface or into the agar were hardly different (data not shown). *L gasseri* CRL1259 and *L johnsonii* CRL1294 did not grow when they were included into the MRS agar plates while none of the six tested lactobacilli were able to grow in this media when they were spread on the surface. For those strains that were able to grow in MRS and LAPTg agars, the diameters of the inhibition halos were wider in MRS than in LAPTg agar for most of the antibiotics tested, as shown in Table 1.

In order to know whether LAPTg or MRS agar was appropriate to be used as a base medium in a standardized method for *Lactobacillus*, the effect of antibiotics on an NCCLS selected type strain inoculated in this medium was evaluated. If the halos for the type strain in LAPTg or MRS agar were of the same diameters to those obtained in Müller Hinton agar, it would suggest that the disc diffusion method could be performed in LAPTg or MRS with NCCLS reference strain. *S aureus* ATCC25922 was inoculated in LAPTg and MRS agar and the diameters of the halos obtained with antibiotic discs were compared to those of Müller Hinton. It was observed that *S aureus* ATCC25922 was able to grow on LAPTg and MRS agars. However, the diameters of the halos were different to those published by the NCCLS for Müller Hinton. The diameters obtained in a Müller Hinton, MRS, and LAPTg agar are shown in Table 2.

### MICs

Considering that the six *Lactobacillus* strains were able to grow in LAPTg, this medium was selected to study the MICs. LAPTg agar or broth was employed and the obtained results are shown in Tables 3 and 4. All the tested lactobacilli were able to grow at elevated concentration of metronidazole (> 1000 *μ*g/mL). They were also able to grow at high concentration of streptomycin (50–100 *μ*g/mL), kanamycin (100–500 *μ*g/mL), quinolones (norfloxacin, 250–1000 *μ*g/mL, and ciprofloxacin, 10–100 *μ*g/mL), chloramphenicol (250 *μ*g/mL), cephalosporins (ceftriaxone, 100 *μ*g/mL; ceftazidime,100 *μ*g/mL), and aztreonam (100 *μ*g/mL). For the other antibiotics assayed, the susceptibility depended on each particular strain. *L johnsonii* CRL1294 and *L paracasei* CRL1289 did not grow at concentrations of 1 *μ*g/mL of novobiocin and vancomycin, but were able to grow at higher concentrations of almost all the other antibiotics (> 100 *μ*g/mL). *L acidophilus* CRL1266 and *L salivarius* CRL1328 were able to grow at 10 and 1000 *μ*g/mL of vancomycin, respectively.

## DISCUSSION

In this paper, the antimicrobial susceptibility of six probiotic vaginal *Lactobacillus* strains was studied. The knowledge of the antimicrobial susceptibility or resistance is of interest to predict the behavior of an exogenously applied probiotic formula in patients subject to any type of chemotherapy, as well as to consider the concomitant use of the probiotic and antibiotics for the restoration of the normal urogenital flora. On the other side, antimicrobial susceptibility of exogenously applied microorganisms needs to be known for treating eventual collateral effects [19–22]. In this regard, the performance of antimicrobial susceptibility testing may be considered as both a necessary selection criterion for probiotic cultures and an effective guide for specific antimicrobial therapy [23].

Up to date, a standardized method to study the antimicrobial susceptibility of microorganisms belonging to the genus *Lactobacillus* has not been published, probably because they have been considered as “GRAS” for the FDA (Food and Drug Administration, USA) [24]. The available standard techniques and the guidelines for the disc diffusion method have been provided by the NCCLS only for selected aerobic and anaerobic bacteria or yeasts related with laboratory clinical diagnostic. However, many researchers have developed modifications of the semiquantitative disc assay for lactobacilli [19, 25–28]. Different base media and type strains have been employed but reference data are still not available. The E-test (AB Biodisk) has also been used and recommended as an easy diffusion test but modifications of the original protocol had to be introduced for lactobacilli [23, 29].

In the present paper, the conventional methodology described by Bauer et al [18] was first applied. Müller Hinton base medium was employed to test the effect of the antibiotics routinely used for the treatment of urinary tract infections (UTIs) on *Lactobacillus* strains. As previously described by other researchers [30], the growth of lactobacilli in Müller Hinton was poor and irregular, and it was not possible to measure the diameter of the inhibition halos. When LAPTg was employed instead of Müller Hinton, the growth was optimum while in MRS it was appropriate only for some *Lactobacillus* strains but not for all of them. The last observation is coherent with the composition of these two media. LAPTg has a wider variety of nutrients and allows the growth of lactobacilli under aerobic or microaerophilic conditions, while MRS as well as LBS [31] seems to be more appropriate for microaerophilic or anaerobic growth (data not shown).

According to our results, the growth of vaginal lactobacilli in LAPTg and MRS agars was homogeneous and the inhibition halos were clearly defined (except for *L gasseri* CRL1259 and *L johnsonii* CRL1294 which were not able to grow in MRS under microaerophilic incubation). Charteris et al [23, 32] have also used MRS for the disc diffusion and the *E*-test under anaerobic incubation conditions in both cases. Based on size of the halos, the mentioned authors have classified the microorganisms into susceptible, moderate susceptible, and resistant. However, the reasons by which they consider the published ranges for the susceptibility category are not explained. Considering that the size of the halos depends on the diffusion media [33], reference data obtained in the same media are supposed to be employed for categorization purposes. Other examples of the use of different base media are the publications of Bayer et al [25] that have used Müller Hinton supplemented with yeast extract and *L*-cysteine (0.2% and 0.05%, resp), Felten et al [26] who have employed Müller Hinton with 5% of sheep blood, and Klein et al [19] who have used the same base media with horse blood (3%). More recently, Klare et al [28] proposed a mixed formulation of Iso-Sensitest broth and MRS with or without supplementation with L-cysteine and Delgado et al [27] the use of MRS.

In order to know if LAPTg or MRS could substitute Müller Hinton as a standard medium, the size of the halos obtained with a closely phylogenetic-related type microorganism, *S aureus* ATCC29213 was determined. Different publications have cited the use of related type strains for this type of studies. Klein et al [19] have reported the use of *Enterococcus* and Felten et al [26] the use of *Staphylococcus* strains. In this study it was observed that the diameters of the inhibition halos for *S aureus* ATCC 29213 in LAPTg or MRS were different to those obtained in Müller Hinton. These observations confirm that the characteristic of “susceptible” or “resistant” defined by NCCLS for assays performed with type strains in Müller Hinton agar cannot be considered when other media are being employed.

The MICs values obtained were dependant on the lactobacilli under consideration as it has also been reported by Danielsen and Wind [30]. Most of the strains have been found to be resistant to high concentrations of chloramphenicol, aztreonam, norfloxacin, ciprofloxacin, ceftazidime, ceftriaxone, and metronidazole. Susceptibility to other antibiotics (rifampicin, erytromicin, novobiocin, vancomycin, ampicillin, tetracycline, clarithromycin, imipenem, and cefotaxime) depended on each particular *Lactobacillus* strain. On the other hand, no correlation had been obtained with the disc diffusion method and the MICs results.

Resistance or susceptibility to vancomycin has deserved a special consideration in terms of classification of lactic acid bacteria, mainly for lactobacilli associated with human infections or isolated from food [26, 34–36]. Hamilton and Shah [37] have used the susceptibility to vancomycin as an aid to identify *Lactobacillus* species. Simpson [35] and Felten et al [26] have associated sensitivity to vancomycin with the *Lactobacillus* acidophilus group or those originally called “Thermobacteria” while Simpson [35] has observed resistance to vancomycin in lactic acid bacteria belonging to the “Betabacteria” group. However, Klein et al [19] and Griffiths et al [20] have reported resistance to vancomycin in different *L acidophilus* strains isolated from clinical samples. According to the results obtained in this work, 4 of 6 lactobacilli were able to grow at concentrations lower than 1 *μ* g/mL of vancomycin. *L crispatus* and *L salivarius*, both homofermentatives (Thermobacteria), were able to grow at vancomycin concentrations higher than 10 and 1000 *μ*g, respectively.

Metronidazole and clindamycin are the most commonly used antibiotics for the treatment of bacterial vaginosis. Candidate probiotic *Lactobacillus* strains were able to grow at high concentrations of metronidazole and clindamycin, except for *L acidophilus* CRL1251 and *L salivarius* CRL1328 that did not grow at concentrations as low as 0.1 *μ*g/mL of the last antibiotic. These results suggest that selected strains could be used for a restoration therapy together with the antimicrobial bacterial vaginosis treatment. Simoes et al [9] have also studied the effect of metronidazole on the growth of vaginal lactobacilli. These authors have observed partial and complete inhibition at concentration above 1000 *μ*g/mL while they have reported a stimulating effect at concentrations between 128 *μ*g/mL and 256 *μ*g/mL. Carlstedt-Duke et al [38] have observed a low effect of clindamycin on lactobacilli when employing this antibiotic simultaneously with the lactic acid bacteria to restore the normal flora of the gut of rats.

Antimicrobial resistance of candidate probiotic lactobacilli was found to be not associated with extra chromosomal elements, as plasmids were not found in the strains, by applying the technique of Maniatis et al [39] (data not shown). This observation would indicate a low probability of antibiotic resistance transmission to pathogenic microorganisms. However, other different methods should be tested to confirm the absence of plasmids, mainly considering that *L salivarius* CRL1328 is an aggregating strain able to produce bacteriocins, both characteristics frequently associated with extra chromosomal DNA [40, 41].

More studies must be undertaken to define the adequate and standardized method to study the antimicrobial susceptibility of the *Lactobacillus* genus. The use of LAPTg and MRS as base media for the disc diffusion method deserves further studies. However, determination of the MICs is, up to date, the only reliable test to predict the susceptibility or the resistance to antibiotics of *Lactobacillus* strains.

## Figures and Tables

**Table 1:** Diameters of the halos obtained for lactobacilli included into LAPTg and MRS agars and tested with antibiotics employed in ambulatory UTI treatment. TMS: trimethoprim-sulfamethoxazole (25 *μ*g), CEC: cefaclor (30 *μ*g), NOR: norfloxacin (10 *μ*g), NAL: nalidixic acid (30 *μ*g), PMD: pipemidic acid (20 *μ*g), AMN: ampicillin (10 *μ*g), CEF: cephalosporin (30 *μ*g), NIT: nitrofurantoin (300 *μ*g), AMS: aminopenicillin-sulbactam (20 *μ*g). Note: commercial discs do not specify the type of cephalosporin employed.

Strain	*Antibiotic*									

	Media	SXT	CEC	NOR	NAL	PMD	AMP	CEP	NIT	AMS

CRL 1251	LAPTg	23/25	29/31	19/21	17/19	21/23	37/39	39/41	30/30	> 39
	MRS	> 40	> 40	31/33	25/27	30/30	> 40	> 40	> 40	> 40
CRL 1266	LAPTg	20/22	30/34	16/22	18/20	20/24	30/34	30/32	20–26	34–36
	MRS	> 28	> 30	21/23	19/21	17/19	40/40	> 30	> 30	> 28
CRL 1289	LAPTg	30/30	36/36	24/30	20/30	30/34	36/36	30/36	30/30	40/40
	MRS	> 34	> 34	33/35	29/31	29/31	33/35	27/29	21/23	40/40
CRL 1328	LAPTg	26/28	34/34	16/18	11/12	18/22	30/34	36/38	24/26	34734
	MRS	29/31	23/25	27/29	17/19	27/29	37/39	39/41	27/29	35/37

**Table 2:** Inhibition halos for *Staphylococcus aureus* ATCC29213 in LAPT and MRS agars compared to results published for NCCLS reference media using antibiotics for UTI treatment. SXT: trimethoprim-sulfamethoxazole, CEC: cefaclor, NOR: norfloxacin, NAL: nalidixic acid, PMD: pipemidic acid, AMP: ampicillin, CEP: cephalosporin, NIT: nitrofurantoin, SAM: aminopenicillin-sulbactam. Means of the diameters obtained in LAPTg and MRS agar from the assays performed by duplicate are shown. Note: commercial discs do not have the specification of the type of cephalosporin employed.

			Halo diameter (mm)		

SAM	NIT	CEP	AMP	PMD	NAL	NOR	CEC	SXT	

18	20	18	10	21	22	22	26	14	LAPTg
24	34	28	16	24	14	32	36	38	MRS
29–37	18–22	27–31	27–35	NP	NP	17–28	29–37	24–32	Müller Hinton*

*Media recommended by NCCLS^1^. NP: data not published.

**Table 3:** Antibiotic MICs (*μ*g/mL) in LAPTg broth for vaginal *Lactobacillus* strains. STR: streptomycin, KAN: kanamycin, NOR: norfloxacin, NOV: novobiocin, CHL: chloranphenicol, VAN: vancomycin y MTZ: metronidazole. The assays were performed by triplicate.

	MIC (*μ*g/mL)						

*Lactobacillus* strain	STR	KAN	NOR	NOV	CHL	VAN	MTZ

*L acidophilus* CRL 1266	50	100	> 1000	10	250	10	> 1000
*L gasseri* CRL 1259	50	500	> 1000	10	250	< 1	> 1000
*L acidophilus* CRL 1251	50	500	500	10	250	< 1	> 1000
*L paracasei* CRL 1289	50	250	1000	< 1	250	< 1	> 1000
*L johnsonii* CRL 1294	50	250	750	< 1	250	< 1	> 1000
*L salivarius* CRL 1328	100	250	250	< 1	250	> 1000	> 1000

**Table 4:** Antibiotic MIC (*μ*g/mL) in LAPTg agar for vaginal *Lactobacillus* strains. CRO: ceftriaxone, CTX: cefotaxime, CAZ: ceftazidime, CIP: ciprofloxacin, IPM: imipenem, CLR: clarithromycin, TET: tetracycline, OXA: oxacillin, NIT: nitrofurantoin, ERY: erythromycin, CLI: clindamycin, AMP: ampicillin, ATM: aztreonam, RIF: rifampin. The assays were performed by triplicate.

	MIC (*μ*g/mL)						

*Lactobacillus* strain	CRO	CTX	CAZ	CIP	IPM	CLR	TET

*L acidophilus* CRL 1266	100	100	100	> 100	1	1	100
*L gasseri* CRL 1259	> 100	> 100	> 100	> 100	> 100	> 100	> 100
*L acidophilus* CRL 1251	100	100	100	100	10	10	10
*L paracasei* CRL 1289	> 100	> 100	> 100	> 100	> 100	> 100	> 100
*L johnsonii* CRL 1294	> 100	> 100	> 100	> 100	> 100	100	100
*L salivarius* CRL 1328	100	1	100	10	10	1	1

	MIC (*μ*g/mL)						

*Lactobacillus* strain	OXA	NIT	ERY	CLI	AMP	ATM	RIF

*L acidophilus* CRL 1266	> 100	1	100	10	1	100	> 100
*L gasseri* CRL 1259	1	1	1	1	> 100	100	0.1
*L acidophilus* CRL 1251	10	10	1	0.1	1	> 100	0.1
*L paracasei* CRL 1289	100	> 100	100	> 100	> 100	100	> 100
*L johnsonii* CRL 1294	> 100	> 100	> 100	> 100	10	> 100	> 100
*L salivarius* CRL 1328	10	10	10	0.1	1	> 100	0.1
